# Relevance and acceptability of using the Quantiferon gold test (QGIT) to screen CD4 blood draws for latent TB infection among PLHIV in South Africa: formative qualitative research findings from the TEKO trial

**DOI:** 10.1186/s12913-018-3088-8

**Published:** 2018-04-16

**Authors:** Deanna Kerrigan, Carrie Tudor, Katlego Motlhaoleng, Limakatso Lebina, Cokiswa Qomfu, Ebrahim Variava, Sandy Chon, Neil Martinson, Jonathan E. Golub

**Affiliations:** 1 The Johns Hopkins Bloomberg School of Public Health, Department of Health, Behavior and Society, 624 North Broadway Street, HH257, Baltimore, MD 21205 USA; 2 The Johns Hopkins School of Medicine, Center for Tuberculosis Research, Baltimore, MD USA; 3 The University of the Witwatersrand, Perinatal HIV Research Unit, Johannesburg, South Africa

**Keywords:** TB, HIV, QGIT, TST, Qualitative methods, South Africa

## Abstract

**Background:**

Tuberculosis (TB) is the leading cause of mortality among people living with HIV (PLHIV), despite the availability of effective preventive therapy. The TEKO trial is assessing the impact of using a blood test, Quantiferon-TB Gold In-Tube Test (QGIT), to screen for latent TB compared to the Tuberculin Screening Test (TST) among PLHIV in South Africa.

**Methods:**

Fifty-six qualitative interviews were conducted with PLHIV and clinical providers participating in the TEKO trial. We explored TB screening, diagnosis, and treatment guidelines and processes and the use of the QGIT to screen for latent TB infection at the time of CD4 blood draw. Thematic content analysis was conducted.

**Results:**

Considerable variability in TB screening procedures was documented due to lack of personnel and clarity regarding current national TB guidelines for PLHIV. Few clinics had started using the TST per national guidelines and many patients had never heard of isoniazid preventive therapy (IPT). Nearly all participants supported the idea of latent TB screening using routine blood drawn for CD4 counts.

**Conclusions:**

Findings indicate that screening for latent TB infection using QGIT from blood drawn for CD4 counts among PLHIV is an acceptable approach to increase latent TB detection given the challenges associated with ensuring systematic latent TB screening in overburdened public clinics.

**Trial registration:**

The results presented here were from formative research related to the TEKO trial (Identifier NCT02119130, registered 10 April 2014).

**Electronic supplementary material:**

The online version of this article (10.1186/s12913-018-3088-8) contains supplementary material, which is available to authorized users.

## Background

Tuberculosis (TB) killed approximately 1.4 million people in 2015 and is the leading cause of mortality among people living with HIV (PLHIV), globally [1]. In 2015, an estimated 400,000 people died of HIV-associated TB, with the majority of these deaths occurring in sub-Saharan Africa [1]. South Africa has among the highest rates of HIV and TB incidence in the world [1]. The provision of isoniazid preventive therapy (IPT) can decrease the incidence of active TB in HIV-infected individuals by 32% [2]. World Health Organization (WHO) recommendations call for ruling out active TB disease in PLHIV prior to providing IPT [3]. Tuberculin skin test (TST) positivity is used to identify those HIV-infected individuals who would benefit from at least 36 months of IPT [3]. The South African Ministry of Health adopted the WHO guidelines in 2014 [4], though with certain caveats regarding duration based on TST results and anti-retroviral therapy (ART) status.

The use of TST, currently the most common screening test for latent TB, remains suboptimal as a positive result does not differentiate between active disease versus prior infection [5]. Moreover, in certain populations false negative results are common, particularly among PLHIV with CD4 counts < 200 cells/mm3 [6]. Several operational barriers have been identified regarding TST in highly burdened health systems and resource-constrained settings. These include: a lack of human resources to administer the test, staff training, and tuberculin shortages [7]. The TST also requires patients to make at least two visits to a health facility, increasing the possibility for losses to follow up.

Newer interferon-gamma release assays (IGRA) have the advantage of using specific *Mycobacterium tuberculosis* antigens, which provide higher specificity [8]. One such assay, the QuantiFERON TB Gold In-Tube (QGIT) test, has been approved and licensed for use in multiple settings, generating debate regarding the possibility of a more effective and widely used screening tool [9]. While IGRA tests are more expensive for a health system than TST in the short term, they may be cost effective and create cost savings in the longer term if they result in significantly better detection and treatment of latent TB with subsequent prevention of TB disease, as has been observed in some settings [10].

Several studies are underway assessing the use of QGIT in clinical care, including the TEKO trial, which is a cluster-randomized trial among 14 clinics in North West Province of South Africa and the parent study for the current qualitative sub-study and analysis. The TEKO (meaning “to test” in Sesotho) study is formally titled, Quantiferon Gold Test for Detecting Tuberculosis (TB) Infection in HIV/AIDS Patients in South Africa and is registered with ClinicalTrials.gov (NCT02119130). The larger TEKO trial will compare the cost-effectiveness of the QGIT collected at the same time as the blood drawn for routine CD4 count among HIV-infected adults, compared with TST only, on the proportion of PLHIV initiated on the appropriate IPT regimen per South African national guidelines [4]. There were 3398 eligible PLHIV enrolled in TEKO and followed for 2 years. Participants were newly HIV-infected, over 18 years of age, and eligible for TST/IPT. The trial started enrollment in 2015 and intervention implementation and evaluation of key outcomes is still ongoing. Follow-up data collection from the TEKO trial will be completed in 2018.

Prior to the TEKO trial, a formative study was conducted to explore attitudinal and operational factors surrounding the implementation of QGIT and TST among patients and providers. Here we present findings from this qualitative exploration, focused on the relevance and acceptability of using QGIT to address barriers to latent TB screening.

## Methods

We conducted formative qualitative research prior to the initiation of the TEKO trial from April to June 2014. The study utilized a cross-sectional design and a total of 56 people participated in one individual, in-depth interview. The sample included 28 PLHIV receiving care in public clinics and 28 of their clinical care providers. As indicated by qualitative research norms, we sought between 20 and 30 participants per population group (e.g. patients and providers) [11, 12]. The 14 clinics included were all general primary health care clinics, which also offered HIV services and were selected to participate in the TEKO trial, which at that point had not yet begun.

Eligibility for the qualitative study included patients who were 18 years of age or more, living with HIV for at least 6 months, and able to converse in English. One female and one male patient participant were interviewed in each participating clinic. These patients were selected by providers based on their knowledge of patients who might be willing and able to narrate their experience with their clinical care. Two clinical care providers that provided TB care were interviewed in each participating clinic. We sought different types of providers involved in providing TB screening and care to patients (e.g. nurses, counselors, and community health workers).

A semi-structured guide was used to facilitate the interviews using open-ended questions Additional file 1. The interviewer freely utilized probes and follow-up questions to create additional depth. Interviews focused on participant’s views and experiences with TB including details of the screening, diagnosis, and treatment process from patient and provider perspectives. All participants were asked their thoughts about the use of the QGIT test to screen blood drawn at the same time as CD4 blood draws for latent TB. Interviews continued until saturation, or redundancy in findings per key themes, was reached, which was determined by readings of individual transcripts as they came in. These readings also helped ensure the quality of the data collection and transcription.

Clinical care providers were approached directly by study staff, while patients were recruited with the assistance of clinic staff. All interviews took place at the clinic where the participant worked or received care in a private room. Written informed consent was obtained from all participants. The interviews took place in English and lasted approximately one hour. Institutional Review Boards of the Johns Hopkins School of Medicine and the University of Witwatersrand in South Africa approved the study.

All interviews were audio recorded and transcribed. Thematic content analysis [13] was conducted coding and synthesizing provider and patient participants’ views and experiences on the key domains outlined above, as well as those that emerged through the course of the interview through the use of case summaries of each interview. Both the first and second author coded the data in parallel and worked together to generate the case summaries. Three salient themes were documented and are presented below on the clinic environment, TB management practices, and perspectives on the use of the QGIT.

## Results

### Characteristics of the sample

The sample of 28 PLHIV was purposively selected to have equal gender representation. The average age of participants was 38.4 (range 20–62 years). Many participants (11/28) had not finished secondary school and a similar number (10/28) were unemployed at the time of the interview. Among those employed, all were blue-collar workers (e.g. domestic worker, driver, construction worker, etc.). On average patients had been living with HIV for 4.6 years, and almost all (26/28) were on ART. Eleven had a history of TB, with 10 diagnosed with TB and HIV at the same time.

Almost all clinical care providers interviewed were women (25/28) and were working as TB focal (12) or professional nurses (11), in addition to 3 counselors, 1 operational manager and 1 community health worker. The majority of these care providers had experience working with TB screening, diagnosis and treatment as part of their duties, although the length, depth and exact nature of this experience varied. The average length of time providers had worked at the clinic was 8.0 years (range 2–25 years).

### Going to clinic as “all-day event”: The clinical care context for TB and HIV

Overall, patients rated the quality of care they received at participating clinics highly. However, all patients interviewed discussed long wait times at the clinics as a major challenge, frustration, and a clear barrier to staying engaged in care and adhering to ART. Most participants stated they arrived at the clinic at around 6 AM, only to be seen often several hours later, and then not leaving until several hours thereafter, well into the afternoon. The general consensus was that going to the clinic, was often an “all day event”, which was perceived as an almost impossible barrier for those who worked outside their home, as well as a significant inconvenience in terms of child care and other domestic responsibilities for those who worked inside their homes. Participants also discussed challenges with distance and transportation to the clinic.

Several participants commented on the overcrowding in the clinic environment, where it felt like there were “thousands of people” waiting and a lack of space for different types of clinical activities (e.g. TB screening). Several participants reported having to return to the clinic multiple times over a short period to collect results, or come back for additional appointments, making adhering to the prescribed care routine even more challenging. There was an overwhelming consensus on the part of patients that these clinics needed extended days and hours and more staff to accommodate their heavy patient caseloads. As one patient relayed:

*“Now we do, some of the people they sleep here outside the gates. So it will be very good and very nice if they can open this clinic for 24 hours. I don’t know how can you do it, as the people of health department, but it would be very good for the life of our nation so that we can live longer. Sometimes you’d find that you have to queue from 4 o’clock in the morning, you go out here 4 o’clock afternoon. You find that twelve hours you’re in the clinic. So it’s like uselessness and what, but it’s about your life. You must stay. So sometimes it’s punishing our mind, we’re hungry, we are thirsty. There’s only this place, the clinic. There’s no shop around. You must travel long so that you can’t find the people behind you being upfront of you. So the queue, that’s the problem about the queue.”*

Among providers, there was some acknowledgement of the concerns described by patients. Their overall perception, however, was that their clinics were working relatively “smoothly” given their high patient loads and that most of the clinic staff that they worked with were dedicated and diligent in their jobs. A few providers did discuss attitudinal issues among providers including negative and stigmatizing views regarding both HIV and TB, as well as a lack of motivation to implement guidelines. They also discussed operational problems at the clinic level including stock outs of TB and HIV medicines and supplies, lack of clinic personnel, and other material resources (e.g. vehicles and tracer teams) to track and retain patients in care.

### Systematically screening for TB in an overburdened clinic environment

Most patients appeared to understand the common symptoms of TB and mentioned signs such as persistent coughing, night sweats, and weight loss. However, many patients reported they were not always specifically asked about TB symptoms at each visit, which was generally every month to pick up their TB and/or HIV medicines.

Providers, on the other hand, were in consensus that it is policy to screen all patients for symptoms of TB at every visit. They did report that due to patient caseload and lack of personnel, screening at every visit did not always happen. Several providers reported that their clinic had just adopted new procedures to improve the efficiency and frequency with which they screen for TB including: recording whether a given patient was screened for TB at that visit by using a standardized national screening tool and placing a copy in the patient’s medical chart, as well as integrating screening into the patients’ care at the clinic, when they were having their vital signs checked, or during the consultation.

Providers indicated that patients with one or more TB symptoms were provided a sputum bottle and asked to produce sputum on the spot and referred to the TB focal nurse. They reported that sputum samples were sent out for processing at the local hospital laboratory using the Xpert® MTB/RIF test. Most providers indicated they received results in 24–48 h. An important challenge described by providers was getting patients to return for results and to start treatment if needed. In turn, most clinics reported having tracer teams in place to be able to find the patients with active TB to come back to the clinic to start treatment.

Based on patient reports, being given a sputum test or asked to “spit into a cup” appeared to be much more common than having received a TST. A few patients mentioned being given a sputum cup to take home, because they were unable to produce sputum at their visit, which they suggested further delayed the TB diagnosis process. Many patients found the sputum process “difficult”. Some clinical providers reported that some patients found it “dirty” and uncomfortable. Others felt that some patients may be worried about perceived stigma related to both TB and HIV if they were asked to provide sputum, as TB is often found among those living with HIV.

Overall, there was a sense that the clinics viewed TB as a priority and that providers were making concerted efforts to improve the screening process. Yet, heavy patient loads and a lack of personnel, continued to inhibit fully operationalizing these efforts.

### Lack of clarity regarding guidelines for TB management among PLHIV

The interviews conducted occurred approximately 6 months after the official release of the South African guidelines on TB management for PLHIV in December 2014, which recommended the use of TST prior to starting IPT. Many providers, however, mentioned that their clinic had not yet received a hard copy of the new guidelines. At the time of the interviews, about half of the providers interviewed reported that their clinic had started implementing TST in adults, whereas others said they were only using it for children under five with a household TB contact. There were some that were simply unclear about what they should be doing. Many discussed having just received guidance that they were supposed to be using TST now, but they were not sure exactly how to operationalize this new procedure. Those that did have experience with TST often had concerns about the ability of the clinic to effectively implement this screening tool. For example, many reported that approximately one third of patients do not return to the clinic in 72 h to read the TST, causing them to have to set the test again and start the process over, however several nurses stated they were not clear on what to do if the patient did not return within 72 h. Regarding the new guidelines, one nurse summarized her clinic’s experience this way:

*It’s a challenge, I don’t want to lie. It’s a big challenge. With recently they told we must do the TST. Yes. And then maybe the challenge …, now we don’t have a challenge as such, because at least they are supplying with the, that Mantoux, the test itself. So you’ll talk to the patient, and then you’ll do the skin test. Most of the time, those we start, just starting, then we newly diagnosed, and then you do every assessment, and then you do the skin test. If the patient agrees, then you do the skin test. And then you ask them to come maybe on Friday. And do it on Thursday, on Tuesday, and ask the patient to come on Friday. And the patient doesn’t pitch up. She comes again in Friday, the other Friday, after two weeks. ..And then that makes the number of the INH, and IPT go down. It’s not like before, when we used to at least with the TB screening, we used to start with IPT. Now it’s not. And then the other challenge maybe there’s, we are not maybe trained, the Sisters are not well trained…that can be another challenge, because maybe they will wait for another one [that is trained]. That Sister is not here, so there’s nobody to do the TST*.

In response to challenges to implementing TST, some providers reported their clinics started adopting innovative practices. For example, in one clinic the management ensured that all nurses were trained on how to place and interpret TST and the clinic rotates a nurse responsible for TST and initiating IPT daily. By having all nurses trained about the TST and IPT it allowed for patients to return to have the TST read at any time and it is not dependent on a particular nurse being on duty. In other clinics, patients coming back to have the TST read were not required to have to wait in line in order to minimize loss to follow up.

### Streamlining screening: Using routine CD4 blood draws to screen for latent TB

Within this context of an often overburdened clinical care environment and a lack of clarity regarding how to interpret and operationalize current TB preventive treatment management guidelines for PLHIV, almost every provider and patient interviewed reported being open to the idea of screening for latent TB at the time of the blood draws for CD4 count. Providers in particular felt this would streamline the latent TB screening process, given that due to heavy patient loads and a lack of personnel, people were simply being missed for screening in a given visit. Providers also discussed the significant challenges they faced in ensuring that their patients returned to have the TST read. There was a consensus that using the blood draws would help overcome many logistical and follow-up problems, indicating its ease compared with TST:

*That would be awesome. Really [laughter], because you take blood, you send it away, and you get your results. It’s not like “come, let’s do this test.” And sometimes it’s itching and irritating for the patients, TST. If it’s just a blood test, send it away, look at the results, start…That would be wonderful. Really, I think it’s amazing if we can do it like that*.

*It might be more accurate, because some of the staff, we’re all trained, but sometimes we’re not sure if they measure it correctly, or if the TST was done correctly, did they take enough, was it inserted sub-dermally? There can come human errors as well. With blood, I think the result is the result. You can see it in the blood. And we do draw the bloods of the patient anyway. We have to do the CD4 count, we have to look at the kidney functions, liver functions, viral loads. If we can do the tuberculosis test with that, it would be great*.

Other providers relayed similar sentiments and enthusiasm, due to the challenges they saw associated with gathering sputum samples. However, this enthusiasm also relayed a misunderstanding on the part of some providers about the differences between the purpose of TST and QGIT, in screening for latent TB, versus the use of sputum and chest X-rays which should be used to establish active TB.

*I will welcome it open handedly you know, because we have quite a few patients if you ask for sputum you send them home with the bottle, they will come back only after two maybe a week or so…and then with some of them, especially if you believe that there might be something you actually send them for chest x-ray. Which I think is more money for the department. So blood will be easier*.

*I like black and white. So if it is done with a blood test, blood has never lied to anybody. If it is done with a blood test, I’m sure it is going to be much easier. Because lots of people you find that they have difficulty with sputum and then the bottles leak or then they put in only spit and not really phlegm and then the bottles got opened along the way. So I, a blood test for me would work much better*.

Patients indicated that while some people are not fond of blood draws, that since the CD4 count blood draw was happening anyway it would be well received. Also, from the patient perspective this would allow for both ensuring that they were screened for TB, as well as potentially avoiding the challenges of taking and returning for the TST, that many patients found time consuming. While there was general consensus on this issue, one patient did relay a concern about timing, as many patients may only have a CD4 blood draw 1–2 times a year. So while this patient thought screening the CD4 blood draw for TB was a good idea, he wouldn’t want that to delay finding out he had TB, as he would want to know that information as soon as possible. This issue was also raised by clinical providers, and a few in turn mentioned that screening the blood for TB could potentially complement, as opposed to replace, the other TB screening methods given that CD4 blood draws occurs generally every 6–12 months. These concerns further exemplify difficulties by both patients and some clinical staff to understand that QGIT is meant to test for latent TB infection rather than active TB disease. Screening for TB symptoms would continue at each patient visit, while the use of QGIT to screen CD4 blood draws could potentially replace use of the TST.

## Discussion

This qualitative study provides important insights into the operational challenges associated with currently recommended TB screening approaches among PLHIV in resource-constrained settings, including use of the TST, as has been documented in prior work^7^. Given the significant burden currently placed on low- and middle-income country health systems, in terms of high patient caseloads, limited personnel, stocks and supplies, long wait times and distances to access care, identifying a more streamlined approach to systematically detect latent TB infection among PLHIV is urgently needed to prevent the leading cause of mortality in this population [14].

Findings from this formative study conducted prior to the start of the now ongoing TEKO trial suggest the likely relevance and acceptability of using the QGIT to screen CD4 blood draws for latent TB infection among PLHIV in rural South Africa.. The approach was viewed as a simple, but potentially powerful, strategy to overcome several logistical barriers to implementing screening for latent TB infection. Yet, both patient and clinical care providers were hesitant to suggest that such an approach should replace other methods, including the use of the TST.

Our qualitative results strongly suggest the need for further dialogue and training regarding the difference between latent and active TB infection and the implementation of the most recent national guidelines on TB among PLHIV in South Africa. Many questions regarding how to interpret and operationalize the guidelines were documented in this exploratory study. As the TEKO trial proceeds, qualitative work will continue to characterize the rollout of the national guidelines and how their adoption in and across clinics may influence the relative effectiveness of a QGIT plus TST versus TST only approach to screening for latent TB infection among PLHIV.

Differences in providers versus patients’ perspectives regarding the daily practices and implementation of current national TB screening approaches underscore the need to engage both groups in future qualitative work regarding their potentially unique experiences in, and perspectives on, the meaning and results of the TEKO trial.

Limitations of the current analysis include the cross-sectional nature of the in-depth interviews conducted, which included only English speakers in the baseline sample, potentially limiting the full range of perspectives specifically among patients. Additionally, patients in this qualitative sample were generally already on ART for some time, whereas TEKO trial participants are newly diagnosed PLHIV. Despite such limitations, our findings revealed strong consistency in terms of patient TB-related experiences in participating clinics and views on how to further strengthen TB management in PLHIV through the use of novel screening strategies such as QGIT.

While this qualitative work was not intended to generate generalizeable results, the common operational barriers to TB screening documented in the literature suggest that QGIT acceptability may be transferable to other low- and middle-income settings.

## Conclusions

Findings indicate that using the QGIT to screen routine CD4 blood draws among PLHIV is an acceptable strategy to increase latent TB detection given the challenges associated with ensuring systematic TB screening in overburdened public clinics.

## Additional file

Additional file 1: In-depth interview guides for patients and care providers. (DOCX 32 kb)
