# Supplementary material for: Relevance and acceptability of using the Quantiferon gold test (QGIT) to screen CD4 blood draws for latent TB infection among PLHIV in South Africa: formative qualitative research findings from the TEKO trial
Source: BMC Health Serv Res. 2018 Apr 16;18:288. doi: 10.1186/s12913-018-3088-8 (PMC5902890; doi:10.1186/s12913-018-3088-8)
Supplement: Supplementary file 1 — In-depth interview guides for patients and care providers. (DOCX 32 kb) [file 12913_2018_3088_MOESM1_ESM.docx]

**In-Depth Interview Guide (Patients)**

*Eligibility:*

HIV-infected patients who have attended the clinic for 3 months or longer

**Clinic Name:** ________________________________________________

**Date of interview:** // **(DD/MM/YYYY) Time:** :

**Participant Sex:** Male Female (circle)

**INTRODUCTION:**

*Thank you for taking the time to speak with me today. As we discussed I am interested in your experiences related to TB screening, diagnosis, and treatment here in this clinic. On behalf of the study team, we appreciate you taking the time to talk with us.*

*There are no right or wrong answers; I am here to learn from you. You may stop the interview at any time and you may choose to not answer any question you would prefer not to answer.*

*Do you have any questions before we start?*

***Background information:***

*First, I would like to start by understanding a bit more about your experiences at this clinic.*

[Please write information in or circle appropriate response]

**D1. What is your age?** ___________________

**D2. What is your highest level of education?** ______________

**D3. What is your occupation?** ____________________________________________________

**D4. How long have you been receiving treatment for HIV at this clinic?** _________

**D5. Are you currently on ARTs?** Yes No (Cirlce)

**D6. How long have you been taking ARTs**? _____________

***Open-ended questions:***

1. What type of services do you receive here at this clinic?

2. How do you feel about the services you receive here at the clinic?

Probe: What do you like? What do you dislike? What could improve?

***Now I would like to talk a bit more specifically about how this clinic screens/tests patients for TB***

4. Have you ever been asked about whether you had TB symptoms by the staff here at this clinic?

Probe: How often do they ask you about TB symptoms?

What do (did) they ask you?

5. Has a nurse or other health care provider at this clinic ever given you a test for TB?

Probe: What type of test did you receive?

6. Please describe that testing/screening process to me?

Probe: Skin test, blood draw, X-ray, spit in a cup, etc.

How was the testing/screening process described or explained to you?

7. How did you find out about your test results?

8. How would you describe the TB testing/screening process at this clinic?

Probe: What is working well with this process?

What is not working well?

9. How can this process of testing/screening for TB be improved?

10. How would you feel about having someone test your blood that is drawn routinely for CD4 testing in order to test for TB?

Probe: Would this be better than a skin test?

How so? Why or why not?

11. Have you heard of isoniazid preventive therapy also called IPT?

Probe: What do you know about IPT and its use?

12. What is your experience with IPT??

Probe: Have you ever taken IPT?

How long (how many months) did you take IPT?

Did you have any side effects to the IPT?

If yes, please describe the side effects you had.

How did you feel when you were taking IPT?

How did this impact taking ART?

***Now I would like to learn more from you about your experiences with TB diagnosis and TB treatment at this clinic.***

13. Have you previously been diagnosed with TB? **IF NO, SKIP TO QUESTION 25**

Probe: If yes, how many times?

How were you diagnosed with TB? What tests were done?

How did you get your results?

Are you currently on TB treatment?

14. Describe what happened from the time a nurse or health care provider told you that you had TB to starting treatment for TB.

Probe: How did you get started on TB treatment?

15. How is the process of linking patients to treatment working here at this clinic?

Probe: What is working well?

What challenges did you experience with the treatment process?

16. How could this process of getting patients on treatment for TB be improved?

17. **If patient has taken TB drugs before:** How did you feel about your ability to manage your TB treatment?

18. Is/was it difficult to adhere to the TB drugs for you? How so?

19. What makes/made it easier or helps/helped you to adhere (stay on) to TB treatment?

20. Did you receive any specific help or support from the clinic or elsewhere?

21. Do you feel like taking TB drugs impacted your ability to take your HIV medicines?

Probe: Tell me more about this…

22. How did you feel taking both TB medicine and your HIV medications (ART?

23. Did you experience any side effects?

Probe: How have you managed those?

24. What else do you think should be done to help PLHIV adhere to both their HIV and TB drugs?

25. **If patient was not previously treated for TB**: How does a patient know when they should be treated for TB?

26. Have you noticed any recent changes to how a patient is diagnosed with TB and gets treatment here at this clinic?

Probe: If yes, what has changed?

How do you feel about these changes?

27. What is your experience with these changes? In your opinion, how have these recent changes worked?

Probe: How have these changes impacted patients at the clinic?

Positively? Negatively?

28. Do you have any other thoughts about how the TB screening, diagnosis, and treatment process or IPT is working at this clinic?

Probe: How it might be improved in the future?

29. Is there anything else you would like to share with me?

*Thank you for your time. We appreciate all your inputs and suggestions.*

**Key-Informant Interview Guide (Providers)**

**Clinic Name:** ____________________________________________

**Date of interview:** // **(DD/MM/YYYY) Time:** :

**Participant Sex: Male Female** (circle)

**INTRODUCTION:**

*Thank you for taking the time to speak with me today. As we discussed I am interested in your experiences related to TB screening, diagnosis, and treatment among people living with HIV. On behalf of the study team, we appreciate you taking the time to talk with us.*

*There are no right or wrong answers; I am here to learn from you. You may stop the interview at any time and you may choose to not answer any question you would prefer not to answer.*

*Do you have any questions before we start?*

***Background information:***

*First, I would like to start by asking you some questions about your background and your role in the clinic.*

[Please write information in or circle appropriate response]

**D1. Participant Job Title:** _________________________

**D2: Participant Training:** (circle)

Doctor Enrolled Nurse

PHC Nurse Other: ______________________

Professional Nurse

**D3. How long have you been a (Doctor/Nurse/other Role)?**__________________

**D4. How long have you worked in this clinic?** __________________

**D5. Type of clinic:** CHC PHC Gateway Clinic (circle)

***Open-ended questions:***

1. Please tell me a bit about what you do here at the clinic: your role and job…

Probe: What are your duties?

Do you have any other duties? Committees? IC, Occ health?

***Now let’s talk about the screening process for TB in your clinic for people living with HIV.***

2. Can you walk me through each step in the process of screening patients living with HIV for TB?

Probe: What happens when someone shows signs and symptoms of TB on the screening test?

What happens if a patient has symptoms of TB?

When does this screening occur?

Who does the screening?

How often are patients screened?

3. How has the screening process changed recently?

Probe: Tell me more about when and how this change occurred?

4. How do you feel the current screening process for TB among PLHIV is working?

Probe: What is working well about this process?

What isn’t working as well in terms of TB screening?

What could be improved in the current screening process?

What other ideas do you have to improve the screening process?

5. What is the clinic policy on skin tests? Is this clinic doing skin tests (TST)?

Probe: Tell me more about who gets tested and when?

What is working well with this process?

What challenges are arising?

***Now let’s talk about the use of isoniazid preventive therapy or IPT in your clinic.***

6. What is the clinic policy on IPT?

Probe: Tell me more about who gets IPT at this clinic?

When does this happen?

7. How does the clinic monitor if patients receive IPT?

Probe: What is monitored?

How often do they have to come in to receive IPT?

How is patient adherence to IPT monitored?

What is the process if a patient misses visits or defaults IPT?

8. How would you feel about testing the blood that is drawn routinely for CD4 testing among PLHIV in order to test for TB?

9. Would this be better than having to do a skin test for example?

Probe: How so? Why or why not?

***Now I would like to hear from you about how the TB diagnosis process works in your clinic.***

10. Can you walk me through the steps from TB screening to diagnosis?

Probe: How does this process occur? Who is involved in this process?

11. What types of samples are collected? How are they processed/analyzed?

12. Have there been recent changes to the diagnostic process recently?

13. How can the process of diagnosis be improved?

***Now I would like to learn more from you about how treatment for TB works in your clinic***

14. Can you walk me through the steps from diagnosis to starting TB treatment for patients in your clinic?

Probe: Once someone is diagnosed with TB – how do they receive or start

treatment? Get registered as a patient, etc.

15. How do you make sure a patient who has TB gets started on treatment?

Probe: What happens if a patient does not come for treatment? How do you follow

up?

16. Have there been recent changes to these treatment procedures?

17. How can the process of linking patients to treatment be improved?

18. How do you think TB treatment impacts a patient’s adherence to HIV medication/ART?

19. Does TB treatment make it easier or more difficult for patients to take ART? If yes, how?

20. What would make adherence to both TB and ART easier for PLHIV here?

Probe: How can we help patients manage taking both of these regimens?

What support systems can help patients adhere to both TB/HIV treatment?

***Now I would like to hear about your experiences regarding training you have received on TB.***

21. I would also like to understand more about the training and support that you have received regarding TB screening, diagnosis and treatment?

Probe: Can you tell me a bit about the training you have received?

22. What additional training and support would be helpful to you?

23. Do you have any other thoughts about how the TB screening, diagnosis, and treatment process is working and/or how it might be improved in the future?

Is there anything else you would like to share with me?

*Thank you for your time. We appreciate all your inputs and suggestions.*

End time: :
